# Improved differentiation of prostate cancer using advanced diffusion models: a comparative study of mono-exponential, fractional-order-calculus, and multi-compartment models

**DOI:** 10.1007/s00261-024-04684-z

**Published:** 2025-02-18

**Authors:** Yongsheng He, Xuan Qi, Min-Xiong Zhou, Mengxiao Liu, Hongkai Yang, Wuling Wang, Bing Du, Shengdong Nie, Xu Yan

**Affiliations:** 1 Ma’anshan People’s Hospital, Ma’anshan, China; 2 Shanghai University of Medicine & Health Sciences, Shanghai, China; 3 University of Shanghai for Science and Technology, Shanghai, China; 4 Siemens Healthineers (China), Pudong, China; 5 Wannan Medical College, Wuhu, China; 6 Shandong First Medical University, Tai’an, China

**Keywords:** MRI diffusion imaging, Prostate cancer, Diffusion models, Fractional-Order-Calculus, Multi-Compartment

## Abstract

**Purpose:**

This study aims to compare the performance of mono-exponential (Mono), fractional-order-calculus (FROC), and multi-compartment (MC) diffusion models in differentiating prostate lesions, including benign prostatic hyperplasia (BPH) and prostate cancer (PCa), as well as classifying PCa by clinical significance and risk levels.

**Methods:**

\A prospective study was conducted with 224 men (aged 50–80) undergoing 3 T MR imaging. Regions of interest (ROIs) analyses were performed on quantitative parameters from Mono, FROC, and MC models. These parameters were evaluated for their ability to distinguish BPH from PCa, clinically significant (CS) from clinically insignificant (CInS) PCa, and among PCa risk levels. Group differences were assessed using the Mann–Whitney U test and Kruskal–Wallis test, followed by post-hoc Dunn’s test. ROC curves were plotted, and AUC was calculated. Logistic regression was used for parameter combinations, and performance was evaluated via 1000 bootstrap samples. The correlation between parameter pairs was analyzed. The image quality and PCa detection capability were also evaluated visually.

**Results:**

In distinguishing PCa from BPH, the F1, ADC, and D parameters from the three models achieved high AUCs of 0.92, 0.91, and 0.91, respectively. For differentiating CS-PCa from CInS-PCa, the F2 parameter and the combination of C1 + F2 from the MC model showed the highest AUCs (0.75 and 0.76). In assessing PCa risk levels, F2 and C1 + F2 from the MC model showed the highest AUCs (0.73 and 0.74) for low vs. intermediate-risk PCa. For intermediate vs. high-risk PCa, F1, F1F2, and β + F1F2 from MC and FROC models had the highest AUCs (0.66, 0.66, and 0.71). In addition, ADC was strongly or moderately correlated to D, μ, F1, F1F2, F3, C1 and C3, and not correlated to β and F2. ADC and C1 demonstrated high image quality and strong PCa detection capability.

**Conclusion:**

Advanced diffusion models, particularly the MC model, demonstrated a significant improvement over ADC in differentiating prostate lesions, especially between low and intermediate-risk PCa, between intermediate and high-risk PCa, and between clinically significant and insignificant PCa. Comparable performance was observed in distinguishing BPH from PCa among three models. Moreover, the combination of MC and FROC models further enhanced differentiation accuracy, particularly in the more challenging classifications between intermediate and high-risk PCa, where ADC alone proved inadequate. These results highlight the potential clinical value of MC model and combining MC and FROC models for more precise PCa risk stratification.

**Graphical abstract:**

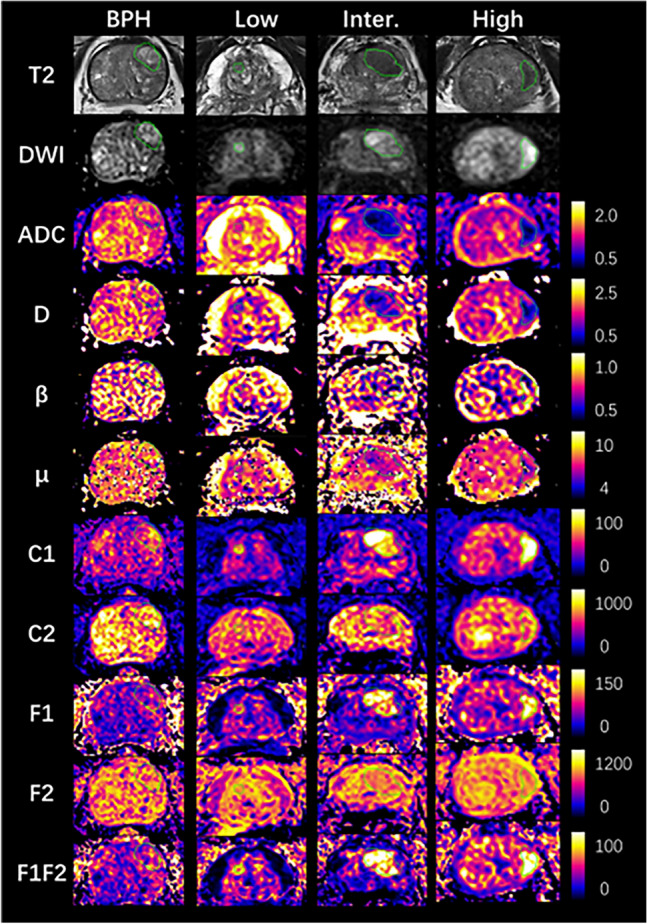

## Introduction

Prostate cancer (PCa) is the second most frequent malignancy in men worldwide [1]. Effective management of prostate lesions relies on accurate detection, differentiation of PCa from benign prostatic hyperplasia (BPH), and precise classification of PCa by different risk levels and clinical significance. BPH, a non-cancerous enlargement of the prostate, typically requires less aggressive management compared to PCa [2]. PCa can be further classified into low, intermediate, and high-risk categories according to the Gleason score (GS), a histological grading system examining the patterns of cancer cells in the prostate tissue [3]. Low-risk PCa (GS 6), often referred to as clinically insignificant prostate cancer (CInS-PCa), might be managed with active surveillance, while intermediate-risk (GS 7) and high-risk (GS ≥ 8) PCa, referred as clinically significant prostate cancer (CS-PCa), often require more aggressive treatments [3].

Multiparametric MRI (mpMRI) has become a crucial tool in identifying suspicious prostate lesions for targeted biopsy [4–10]. Compared to PSA, which is commonly used for initial screening, studies have shown that mpMRI can significantly enhance accuracy in differentiating between BPH and PCa, as well as across different grades of PCa [11–13]. This improved precision helps reduce unnecessary biopsies and overdiagnosis. Despite its wide use, mpMRI, which includes T2-weighted imaging, diffusion-weighted imaging (DWI), and apparent diffusion coefficient (ADC) maps, showed limited accuracy in distinguishing CS-PCa across various risk levels [14]. The ADC, derived from a conventional mono-exponential (Mono) model, measures an overall diffusion metric of water motion, thus missing detailed microstructural information from multiple prostate tissues. Previous studies reported that ADC cannot accurately predict PCa from low-grade to higher-grade, where a mixture of poorly differentiated tissues often exists [2, 6, 15, 16].

To overcome these limitations and better capture tissue complexity, several advanced diffusion models have been introduced, such as diffusion kurtosis imaging (DKI) [17, 18], intravoxel incoherent motion (IVIM) [19, 20], fractional-order-calculus (FROC) [21, 22], and multi-compartment (MC) [23, 24] models. DKI quantifies non-Gaussian water diffusion, reflecting tissue complexity and heterogeneity, while IVIM, more specifically, distinguishes between perfusion-related and pure diffusion signals.

The FROC model introduces two additional parameters β and μ as well as diffusion coefficient D to quantify heterogeneity and mean free motion path of water molecules within a voxel. Recent studies have shown that the FROC model outperforms the Mono, DKI, and IVIM models in differentiating CS-PCa from CInS-PCa in the transition zone[16, 25]. Unlike the FROC model, the MC model separates the signal from a voxel into multiple diffusion compartments, with each compartment potentially reflecting distinct biological microstructures [24]. Recent studies indicate that the MC parameter, particularly the signal from the restricted diffusion compartment, effectively excludes diffusion signals from benign prostate tissue. This improves its ability to distinguish between normal tissue and PCa, as well as CS-PCa from CInS-PCa [15, 26, 27].

However, despite these advancements, no single model has been conclusively reported to outperform the others across all lesion classifications. It is also unclear whether combining parameters from different models provides added diagnostic value. For instance, FROC parameters may reflect overall diffusion heterogeneity, while MC parameters capture specific tissue compartment signals. Exploring these combinations may yield better performance than relying on a single model alone, thus supporting more nuanced clinical decision-making.

This study, therefore, aims to evaluate the performance of Mono, FROC, and MC models—both individually and in combination—in distinguishing PCa from BPH, CS-PCa from CInS-PCa, and among various PCa risk levels. Our goal is to determine whether one model consistently outperforms the others or whether combining diffusion parameters enhances diagnostic accuracy in PCa management. By integrating these advanced diffusion parameters, we aim to improve prostate cancer classification and ultimately enhance clinical decision-making.

## Materials and methods

### Study population

This study was approved by the Institutional Review Board of hospital’s affiliated university (IRB-2021006008). A total of 249 men, aged 50–80 years, underwent pelvic MRI between November 2021 and October 2023. These participants were selected based on the availability of complete clinical and histopathological data, and the Gleason scores (GS) of PCa ranged from 6 to 9. Twenty-five Men were excluded if they had undergone prior endocrine therapy, radiotherapy, or surgical interventions, or if their MR images were affected by artifacts. Ultimately, 129 patients with BPH and 95 patients with PCa were identified through both mpMRI and biopsy data, including 14 with GS 6, 56 with GS 7, and 25 with GS ≥ 8.

## Image acquisition

MRI data were acquired using a 3 T scanner (MAGNETOM Prisma, Siemens Healthineers, Erlangen, Germany) equipped with an 18-channel body coil and a 32-channel spine coil. Each patient underwent a high resolution T2-weighted sequence and multi-b-value diffusion sequence. Diffusion data were acquired using a Zoomit single-shot diffusion sequence with the following scan parameters: repetition time 3500 ms; echo time 74 ms; b-values of 0, 50, 500, 1000, 1500, 2000, and 2500 s/mm^2^, collected in three different directions and geometrically averaged, with diffusion gradient pulse width ( δ): 32.2 ms and gradient lobe separation (Δ): 47 ms; slice thickness 3 mm, covering 23 slices; field of view 200 × 109 mm^2^; voxel size 0.9 × 0.9 mm^2^; bandwidth of 1568 Hz/Pixel. The total collection time of diffusion sequence is 6 min and 18 s. Inline dynamic field correction was applied to eliminate image distortion caused by eddy currents.

## Image analysis

The prostate mpMRI data were reviewed by two radiologists, each with over 10 years of experience, and lesions were identified according to PIRADS criteria. To ensure image quality, the PIQUAL system is used to score prostate multiparametric MRI data with five levels from 1 to 5, namely non-diagnostic, insufficient, sufficient, good quality and sufficient, excellent quality to diagnosis PCa. Regions of interest (ROIs) were delineated within the borders of the lesions on DWI images with a b-value of 1500 s/mm^2^ by one radiologist with over 10 years of experience and validated by another radiologist with similar expertise. The ROIs were outlined using the open-source, multi-platform software ITK-SNAP (version 4.0.1, www.itksnap.org), and then copied to all quantitative parameter maps to calculate the mean values of the ROIs. Subsequently, the ROIs were matched with pathological findings from transrectal ultrasound-guided prostate biopsies by two physicians, each with over 10 years of experience in urogenital MRI diagnosis.

For each patient’s data, quantitative parameters of diffusion models were acquired using the BoDiLab software (MRStation, Zhongying, Chengdu, China), including the Mono, FROC, and MC models. The preprocessing of diffusion data involved two steps: background removal and image smoothing. Background removal was performed based on the baseline image at b = 0 by setting a grayscale threshold according to the signal in the background area. Image smoothing was achieved using 3D Gaussian filtering with a smoothing kernel sigma of 1.25 to improve the signal-to-noise ratio (SNR).

The preprocessed diffusion data were subsequently used to fit diffusion models and generate quantitative maps. In the Mono model, ADC value was fitted using the following equation:

1 $$ S(b) = S(0)\,\exp ( - b \cdot \,ADC) $$

Where S(0) is the baseline signal at b = 0. The b-value at 50, 500 and 1000 s/mm^2^ were used in the fitting process, which is a common b-value range in previous studies [26, 28] and reflects the average diffusion coefficients of intra- and extra-cellular space Lower b-values were started from 50 s/mm^2^ to eliminate the influence of perfusion signal.

For the FROC model, a Levenberg–Marquardt nonlinear fitting algorithm was applied to the following equation:

2 $$ S(b) = S(0)\,\exp \left\{ { - D \cdot \mu^{2(\beta - 1)} \cdot (\gamma G\delta )^{2\beta } \cdot \left( {\Delta - \frac{2\beta + 1}{{2\beta - 1}}\delta } \right)} \right\} $$

where G and δ are the amplitude and pulse width of diffusion gradient, respectively, and Δ represents the gradient lobe separation. The fitted parameters D, β and μ reflect the diffusion coefficient, diffusion heterogeneity, and the mean free path of water molecules within the diffusion environment. The two-step algorithm was used [22], the initial value of D was first estimated by a mono-exponential fit using data acquired at b-values ≤ 1000 s/mm^2^, after which β and μ were calculated using Eq. (2) and all DWI data.

In the MC model, the overall diffusion signal is represented by a linear sum of exponential decays, each representing tissue compartments with different diffusion coefficients. The equation is as follows:

3 $$ S(b) = \sum\limits_{i = 1}^{N} {} C_{i} e^{ - bDi} $$

where C_i_ represents the signal contribution of each compartment, D_i_ represents the diffusion coefficient of the corresponding compartment and N represents the number of compartments.

A In this study, a four-parameter model was adopted (N = 4), which has been validated in previous research as the optimal configuration for compartment number and corresponding D values. The optimal D values of four compartments were set as 5.2 × 10^–4^, 1.9 × 10^–3^, 3.0 × 10^–3^, and 3.0 × 10^–2^ mm^2^/s, representing restricted diffusion, hindered diffusion, free diffusion, and vessel flow components, respectively [15]. Nonnegative Linear fitting was used to calculate C_i_ with all b-value data at each voxel. The signal fraction of each compartment F_i_ were also calculated by normalizing C_i_ with S(0), namely Fi = Ci / S(0), to eliminate the influence of T2, proton density, and scanner-dependency.

## Statistical analysis

Prostate lesions were first classified into BPH and PCa groups. The PCa group was further divided based on clinical significance into CInS-PCa (GS 6) and CS-PCa group (GS ≥ 7). The Mann–Whitney U test was performed for these two-group comparisons. Subsequently, the PCa lesions were categorized into low (GS 6), intermediate (GS 7), and high-risk (GS ≥ 8) groups according to NCCN risk guidelines [3]. Comparisons among these risk groups were conducted using the Kruskal–Wallis test, followed by post-hoc Dunn’s test. For each classification, the mean and standard deviation were also calculated.

Afterwards, the prostate lesions were organized into four comparison categories: low vs. intermediate risk, intermediate vs. high risk, CS-PCa vs. CInS-PCa, and BPH vs. PCa. The performance of each quantitative parameter from Mono, FROC, and MC models, as well as their combinations, in differentiating between lesion groups was plotted on receiver operating characteristic (ROC) curves. The combination of two diffusion parameters from different models was evaluated using logistic regression under the hypothesis that each diffusion model may offer complementary information for prostate lesion classification. While we initially focused on evaluating specific pairs of parameters to avoid overfitting problem. The area under the curve (AUC) was calculated, then the parameters and their combinations with the top five AUC values were presented. To improve the robustness, classification performance was assessed via 1000 bootstrap samples with patient-level resampling to yield the mean and 95% confidence interval of the AUC. All statistical analyses and machine learning modeling were performed in Python 3.8 using scipy.stats and scikit-learn library.

Finally, a correlation analysis among these diffusion parameters pairs were performed. The Pearson correlation coefficient was calculated.

## Visual analysis

A visual analysis was conducted to assess the image quality and capability of PCa detection for diffusion parameter maps. The diffusion parameters that exhibited strong PCa detection and grading capabilities, based on prior statistical analysis, were evaluated by two independent, blinded expert readers. A 4-point scoring system (insufficient, sufficient, good, excellent) was used for both image quality and lesion detection. The kappa coefficient was calculated to assess inter-observer agreement.

## Results

### Group comparisons

**Fig. 1:**
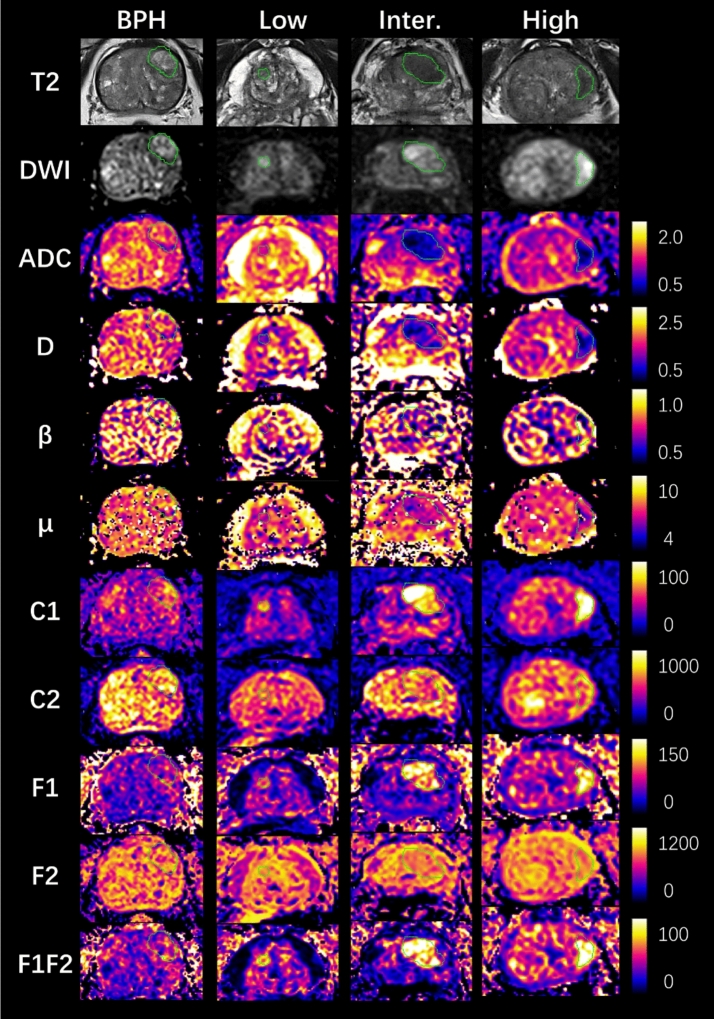
Example parameter maps from the mono-exponential (Mono), fractional-order-calculus (FROC), and multi-compartment (MC) models for BPH and low, intermediate, and high-risk PCa. Units: D (μm^2^/s), μ (μm), other parameters are dimensionless. BPH: 78-year-old male. Low-risk: 78-year-old male with GS 6 (3 + 3) PCa. Intermediate-risk: 70-year-old male with GS 7 (4 + 3) PCa. High-risk: 80-year-old male with GS 8(4 + 4) PCa. The green line showed the border of PCa

**Fig. 2:**
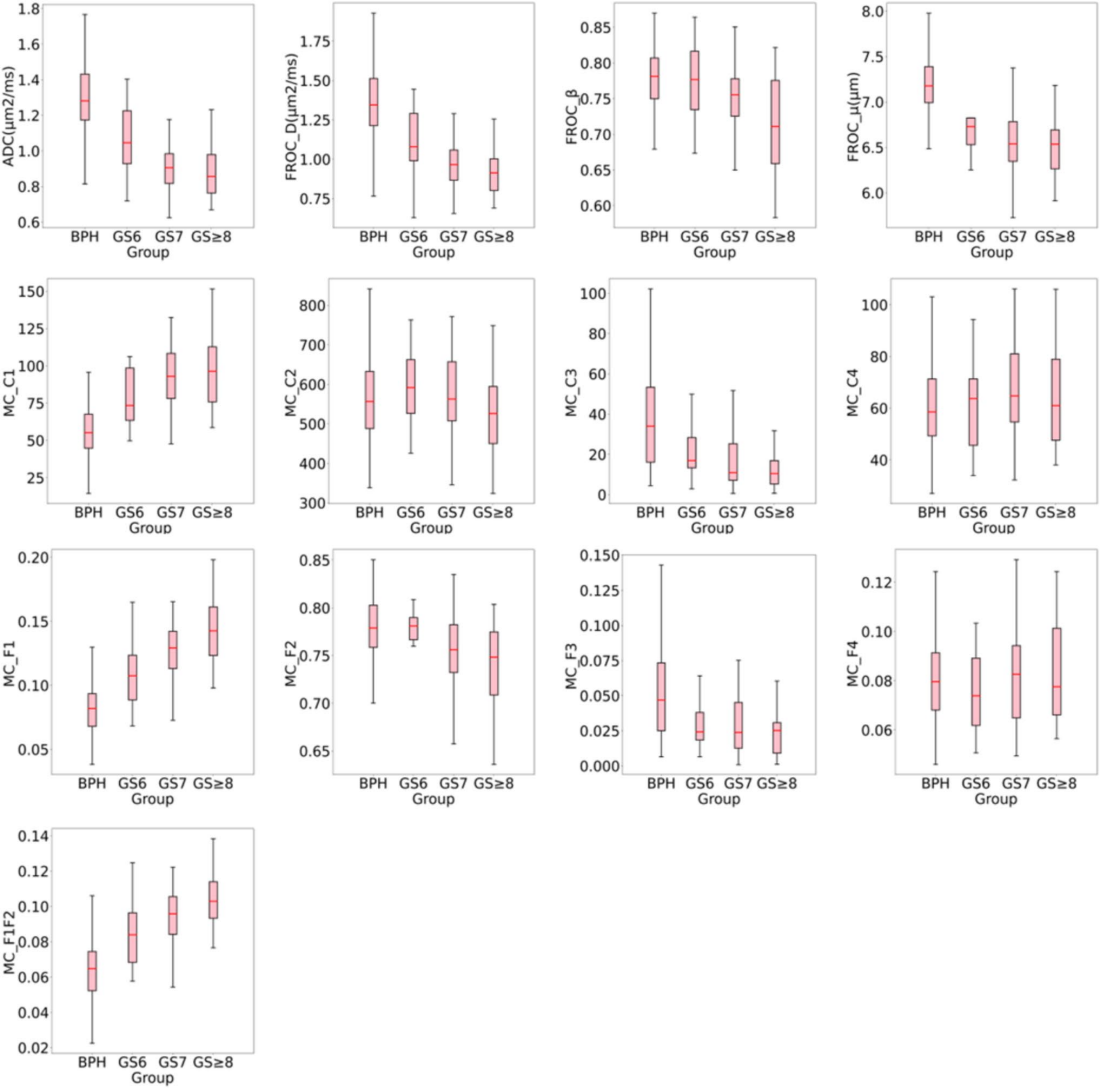
Boxplots show distribution of diffusion maps from Mono, FROC and MC models for BPH and PCa with different Gleason scores (GS). Boxes show the 25th and 75th percentiles, as well as the median (red line)

High quality multi-parameter prostate imaging was acquired, with a mean PIQUAL score 4.55. Figure 1 and 2 show the example case of all diffusion parameter maps from BPH, low (GS6), intermediate (GS7), and high-risk (GS ≥ 8) PCa groups, and their value ranges among groups respectively. For the Mono and FROC models, the mean ROI values of ADC and D showed a consistent decrease from the BPH group to the GS ≥ 8 PCa group, with ADC values decreasing from 1.302 to 0.87 μm^2^/s and D values from 1.373 to 0.912 μm^2^/s. Similarly, the mean ROI values of β and μ also showed a continuous decreasing from BPH to the GS ≥ 8 PCa group, with β values decreasing from 0.781 to 0.714 and μ values from 7.166 to 6.195 μm. Notable reductions in μ were observed between BPH and GS6, and in β between GS7 and GS ≥ 8. In the MC model, the mean ROI values of C1, F1, and F1F2 showed a continuous increase from the BPH group to the GS ≥ 8 PCa group, with values rising from 56.688 / 0.081 / 0.063 × 10⁻^3^ to 100.864 / 0.145 / 0.105 × 10⁻^3^, respectively. Conversely, the mean ROI values of C2 and F2 decreased consistently from the GS6 group to the GS ≥ 8 PCa group, from 589.620 / 0.749 to 535.910 / 0.734. However, the C2 and F2 values of BPH group overlapped with those of PCa across all three risk levels. In addition, no clear trend was observed in the ROI means of C3, C4, F3, and F4 from BPH to the GS ≥ 8 PCa, except for notable changes in C3 and F3 between BPH and GS6. Table 1 presents the mean and standard deviation of each diffusion parameter across all four lesion groups.

**Table 1:** Summary of FROC parameter and ADC means of different lesion groups

Lesion Type		BPH	GS 6	GS 7		GS ≥ 8
Mono	ADC	1.302 ±	1.061 ±	0.936 ±		0.870 ±
		0.202	0.201	0.168		0.134
FROC	D	1.373 ±	1.100 ±	0.992 ±		0.912 ±
		0.212	0.223	0.197		0.172
	μ	7.166 ±	6.700 ±	6.496 ±		6.195 ±
		0.366	0.501	0.667		1.163
	β	0.781 ±	0.777 ±	0.751 ±		0.714 ±
		0.044	0.055	0.05		0.071
MC	C1	56.688 ±	78.692 ±	93.138 ±		100.864 ±
		17.229	18.708	24.695		30.749
	C2	561.997 ±	589.620 ±	574.734 ±		535.910 ±
		115.274	97.043	103.029		130.528
	C3	46.832 ±	22.775 ±	23.343 ±		12.242 ±
		50.633	15.759	33.369		9.677
	C4	62.117 ±	60.682 ±	65.951 ±		63.339 ±
		18.172	17.888	18.468		18.479
	F1	0.081 ±	0.110 ±	0.127 ±		0.145 ±
		0.02	0.029	0.025		0.029
	F2	0.774 ±	0.782 ±	0.749 ±		0.734 ±
		0.045	0.027	0.055		0.05
	F3	0.061 ±	0.033 ±	0.036 ±		0.023 ±
		0.052	0.028	0.042		0.015
	F4	0.080 ±	0.075 ±	0.081 ±		0.084 ±
		0.019	0.018	0.019		0.021
	F1F2	0.063 ±	0.085 ±	0.094 ±		0.105 ±
		0.016	0.021	0.018		0.017

GS is Gleason Score. The units of D and ADC is μm2/s; β is dimensionless, and μ is μm

Table 2 presents the significance of paired comparisons for each diffusion parameter. The Kruskal–Wallis test showed significant differences (p < 0.05) of all diffusion parameters among the low (GS6), intermediate (GS7), and high-risk (GS ≥ 8) lesion groups. Post-hoc Dunn’s test of paired comparisons revealed significant differences in ADC, F1, and F2 between GS6 and GS7 PCa. Significant differences were also found in ADC, D, β, C1, C3, F1, F2, and F1F2 between GS6 and GS ≥ 8 groups, and in β, F1, and F1F2 between GS 7 and GS ≥ 8 groups. Additionally, the Mann–Whitney U test demonstrated significant differences between CS and CInS-PCa in ADC, D, β, C1, F1, F2, and F1F2. The Mann–Whitney U test comparing BPH and PCa also showed significant differences in almost all parameters, except for C2, C4, and F4.

**Table 2:** Kruskal–Wallis* Post-Hoc Dunn’s test of low, intermediate, and high risk PCa

Group 1	Group 2	Dunn’s Test Significance (p-value)**												
		ADC	D	μ	β	C1	C2	C3	C4	F1	F2	F3	F4	F1F2
Low Risk (GS 6)	Intermediate Risk (GS 7)	0.030	0.055	0.175	0.171	0.051	0.651	0.126	0.411	0.049	0.011	0.671	0.340	0.112
Low Risk (GS 6)	High Risk (GS ≥ 8)	0.003	0.007	0.064	0.007	0.024	0.117	0.025	0.787	< 0.001	0.002	0.390	0.273	0.003
Intermediate Risk (GS 7)	High Risk (GS ≥ 8)	0.159	0.178	0.373	0.044	0.482	0.106	0.224	0.518	0.028	0.242	0.507	0.737	0.031

Mann–Whitney U Test of Clinically Significant vs. Insigificant PCa and BPH vs. PCa														
Group 1	Group2	Mann–Whitney U Signifiance (p-value)												
		ADC	D	μ	β	C1	C2	C3	C4	F1	F2	F3	F4	F1F2
Clinically Insignificant (GS 6)	Clinically Sigificant (GS ≥ 7)	0.009	0.020	0.105	0.033	0.029	0.412	0.059	0.410	0.006	0.004	0.546	0.222	0.021
BPH	PCa	< 0.001	< 0.001	< 0.001	< 0.001	< 0.001	0.761	< 0.001	0.300	< 0.001	< 0.001	< 0.001	0.876	< 0.001

*Kruskal–Wallis is significance at p < 0.05. ** The p-values of paired comparisons in Dunn’s Test underwent Bonferroni correction for multiple tests

### ROC analysis

**Fig. 3:**
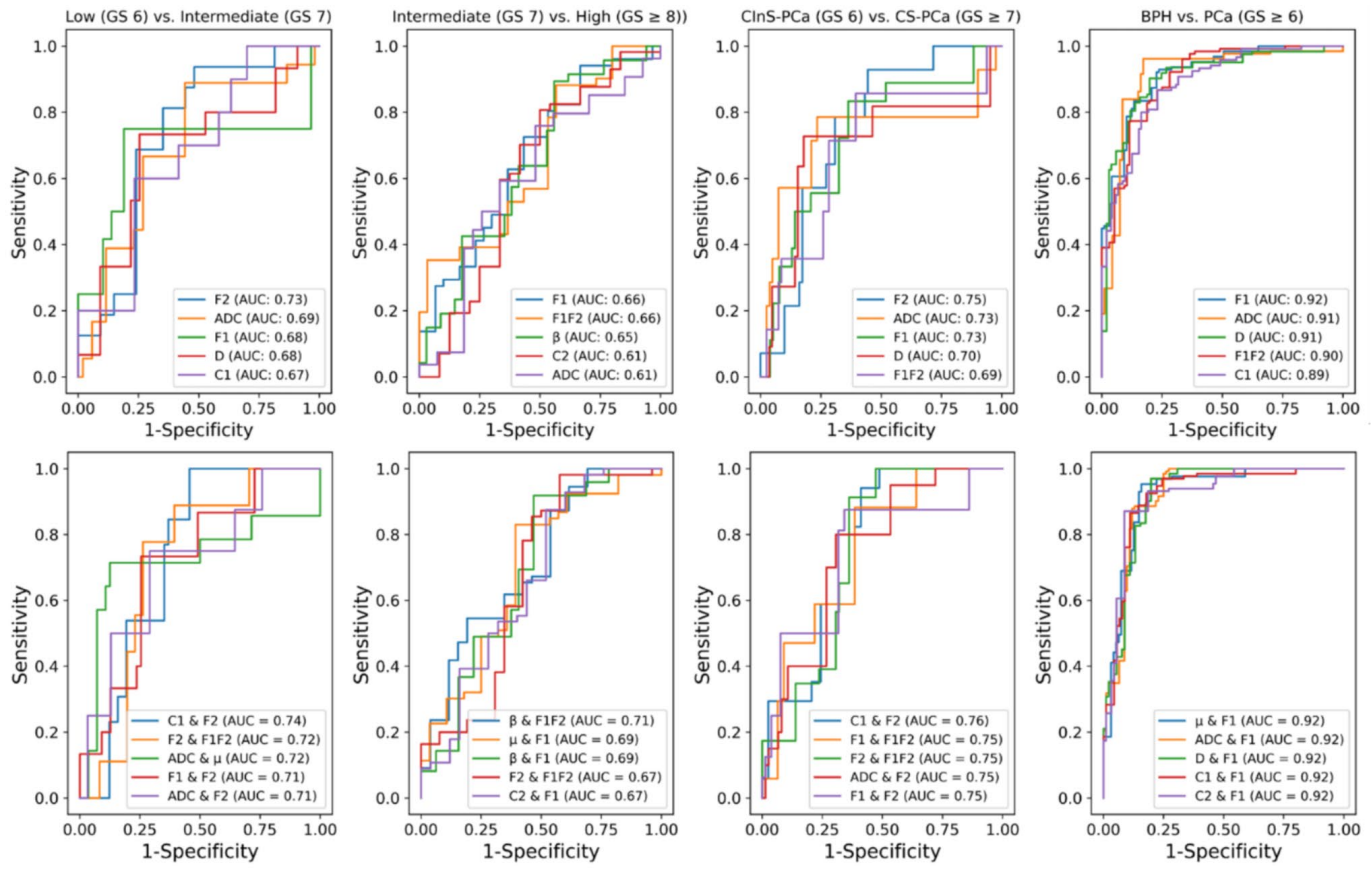
ROC plots of diffusion maps from Mono, FROC and MC models. The mean and the 95% confidence interval of AUC were provided

Figure 3, Table 3 and 4 display the ROC curve and analysis results of the diffusion parameters from the Mono, FROC, and MC models, as well as their combinations. The mean and 95% confidence interval of the AUC for each parameter are shown, along with the AUC for their combinations. For distinguishing PCa from BPH, high AUCs (0.92, 0.91, and 0.91) were achieved by the single variables F1, ADC, and D, respectively. However, combinations involving F1 and one from μ, ADC, D, C1, or C2 did not show further improvement in AUC (0.92).

**Table 3:** ROI analysis results of diffusion parameters in classifying prostate cancer groups

	AUC	5% C.I	95% C.I	sens	spec
*Low (GS 6) vs. Intermediate (GS 7)*					
F2	0.727	0.615	0.834	0.813	0.648
ADC	0.693	0.545	0.836	0.889	0.558
F1	0.682	0.544	0.825	0.750	0.810
D	0.676	0.535	0.821	0.733	0.745
C1	0.673	0.539	0.798	0.600	0.767
C1 & F2	0.735	0.591	0.845	1.000	0.544
F2 & F1F2	0.719	0.562	0.843	0.778	0.738
ADC & μ	0.718	0.562	0.849	0.714	0.875
F1 & F2	0.714	0.564	0.838	0.733	0.745
ADC & F2	0.712	0.542	0.847	0.750	0.710
*Intermediate (GS 7) vs. High (GS ≥ 8))*					
F1	0.664	0.546	0.762	0.725	0.567
F1F2	0.659	0.543	0.769	0.353	0.967
β	0.646	0.528	0.764	0.894	0.441
C2	0.614	0.516	0.727	0.807	0.500
ADC	0.606	0.510	0.723	0.759	0.519
β & F1F2	0.712	0.597	0.817	0.545	0.808
μ & F1	0.692	0.564	0.796	0.830	0.607
β & F1	0.689	0.572	0.800	0.918	0.531
F2 & F1F2	0.669	0.552	0.775	0.982	0.423
C2 & F1	0.667	0.545	0.770	0.875	0.480
*CInS-PCa (GS 6) vs. CS-PCa (GS ≥ 7)*					
F2	0.748	0.632	0.844	0.929	0.556
ADC	0.728	0.577	0.851	0.786	0.765
F1	0.725	0.576	0.849	0.833	0.636
D	0.701	0.554	0.843	0.727	0.821
F1F2	0.691	0.539	0.835	0.857	0.605
C1 & F2	0.757	0.628	0.851	0.941	0.590
F1 & F1F2	0.754	0.614	0.865	0.882	0.615
F2 & F1F2	0.749	0.594	0.863	0.913	0.639
ADC & F2	0.749	0.547	0.866	0.800	0.693
F1 & F2	0.748	0.613	0.868	0.875	0.658

**Table 4:** ROI analysis results of diffusion parameters in differentiating BPH and PCa

BPH vs. PCa (GS ≥ 6)					
	AUC	5% C.I	95% C.I	sens	spec
F1	0.916	0.882	0.947	0.835	0.866
ADC	0.911	0.869	0.943	0.962	0.828
D	0.907	0.866	0.938	0.902	0.802
F1F2	0.903	0.865	0.933	0.773	0.885
C1	0.885	0.849	0.920	0.800	0.837
μ & F1	0.920	0.881	0.950	0.953	0.842
ADC & F1	0.919	0.887	0.950	0.879	0.880
D & F1	0.918	0.882	0.951	0.970	0.802
C1 & F1	0.918	0.882	0.946	0.866	0.889
C2 & F1	0.917	0.881	0.947	0.871	0.913

In the comparison between CS-PCa and CInS-PCa, F2 and the combination of C1 and F2 showed higher AUCs (0.75 and 0.76) than other parameters and combinations. For differentiating PCa risk levels, F2 from the MC model and the combination of C1 and F2 showed the highest AUCs (0.73 and 0.74, respectively) for low vs. intermediate-risk PCa, while F1, F1F2, and the combination of β and F1F2 had the highest AUCs (0.66, 0.66, and 0.71, respectively) for intermediate vs. high-risk PCa.

### Correlation analysis

ADC exhibited strong correlations with D, μ, F1, and F1F2, with correlation coefficients of 0.97, 0.90, -0.95, and -0.96, respectively. Moderate correlations were observed between ADC and C1, C3, and F3, at -0.74, -0.74, and -0.73, respectively. Additionally, β (0.25) and F2 (0.02) showed low correlation with ADC.

### Visual evaluation

**Fig. 4:**
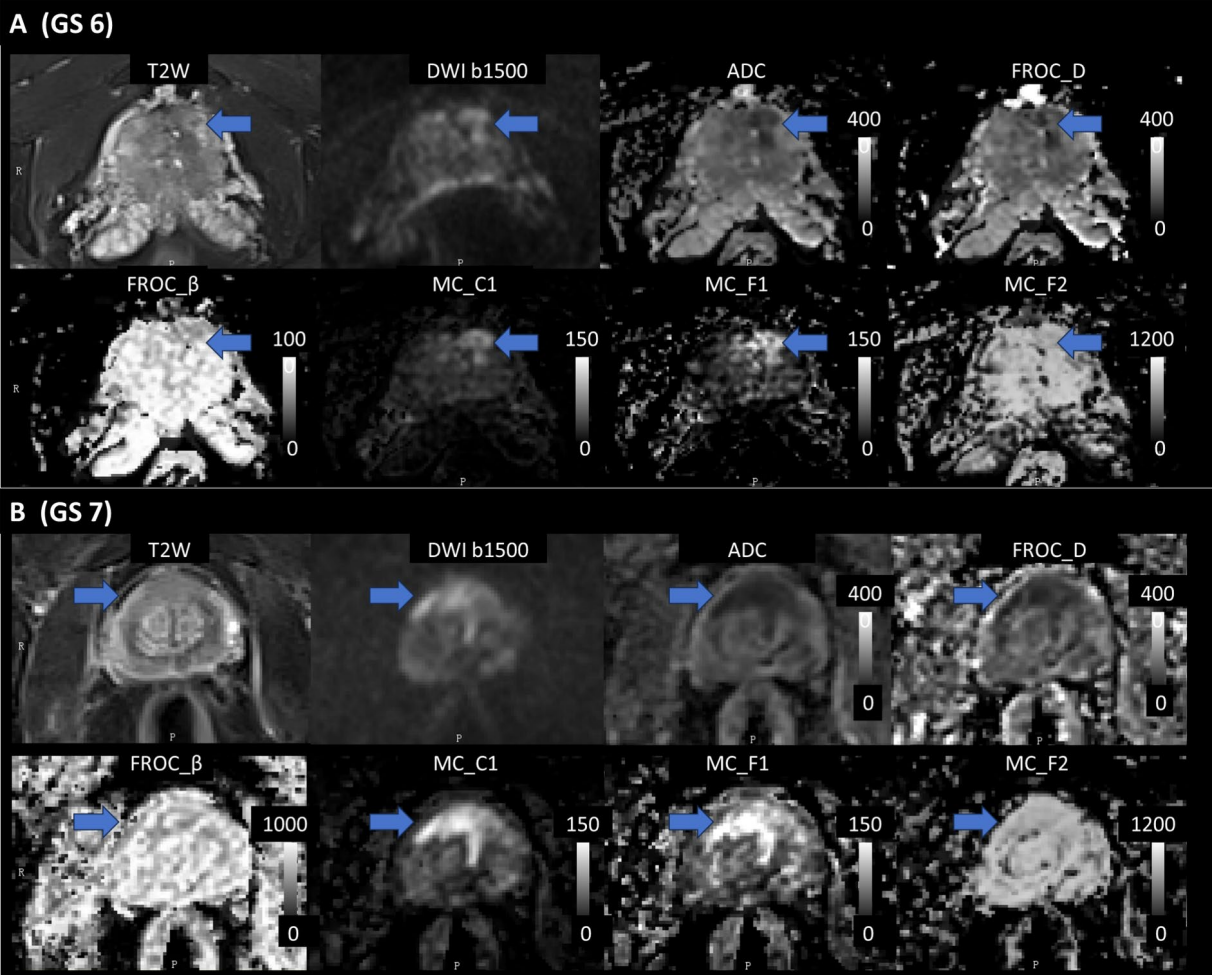
Axial images of two patients with PCa: GS 6 (A) and GS 7 (B). Displayed are the T2-weighted images, DWI images at b 1500, conventional ADC maps, D and β parameters from the FROC model, and C1, F1, F2 parameters from the MC model. The blue arrow indicates the location of prostate cancer (Color figure online)

For image quality assessment, high scores were observed for ADC (3.8), FROC D (3.1), and MC C1 (3.0), indicating good to excellent image quality. Sufficient to good image quality were noted for MC F1 (2.5), F1F2 (2.2), F2 (2.1) and FROC β (2.3), μ (2.1). In terms of prostate cancer (PCa) detection, MC C1 (3.4) and ADC (3.3) demonstrated good to excellent specificity for PCa detection, while FROC D (2.5), MC F1 (2.4), and F1F2 (2.3) exhibited sufficient to good detection capabilities. Parameters such as FROC β and μ showed insufficient detection capabilities, with scores below 2.0. Inter-observer agreement for image quality assessment was good, with a Cohen’s Kappa value of 0.76. In Fig. 4, two patient cases showed high image quality and lesion contrast of ADC, D, C1, where C1 exhibit good suppression of signal from normal prostate tissue.

## Discussion

This study aims to evaluate the performance of multiple diffusion models in distinguishing PCa from BPH, as well as in differentiating between various risk levels of PCa and between CS and CInS-PCa. ROI and ROC analyses were performed on quantitative maps derived from mono-exponential, fractional-order-calculus and multi-compartment models. The results revealed that although ADC showed significant differences in the comparisons of BPH vs. PCa, CS-PCa vs. CInS-PCa, and low vs. intermediate-risk PCa, the FROC and MC models, as well as their combinations, demonstrated superior performance in these comparisons. Furthermore, these advanced models showed significant differences between intermediate and high-risk PCa, where ADC failed to differentiate.

The ADC map from the Mono model, despite its simplicity, is a robust imaging marker for differentiating between BPH and PCa, as well as between CS-PCa and CInS-PCa [6, 28, 29]. However, it faces challenges to distinguish between intermediate and high-risk PCa [16, 26, 30]. Similarly, our study found that ADC significantly differentiated PCa from BPH and CS-PCa from CInS-PCa, but failed to distinguish between intermediate and high-risk PCa. This limitation may be due to the inherent heterogeneity and the mixture of poorly differentiated tissues in higher tumor grades [31]. ADC average the diffusion properties from different tissue components [16], leading to overlapping values that obscure specific information regarding PCa grades.

Previous studies on the FROC model have demonstrated its significant potential in differentiating between various risk levels of PCa based on its β and μ parameters [16, 25]. Similarly, our study showed that β outperformed ADC in distinguishing changes between GS 7 and GS ≥ 8 [16], and combined β with F1F2 from MC model showed highest AUC in this comparison. This is due to β’s ability to capture subtle variations in tissue heterogeneity and microstructural complexity, which are crucial for accurate risk stratification of PCa. However, different from other studies [16, 25], our results indicated that β alone did not perform better than ADC in distinguishing between low and intermediate-risk PCa, and between CS-PCa and CInS-PCa. This discrepancy may be attributed to the influence of b-value choice and signal-to-noise ratio in model calculation [32].

The MC model consistently showed the highest performance across all lesion groups, which may stem from its capability to capture the tissue microstructure variations associated with different Gleason scores. The C1 and F1 parameters showed a continuous increase from BPH to high-risk PCa, corresponding to an increase in restricted diffusion components. It reflects higher cellular density and greater nuclear-to-cytoplasmic ratios seen in more aggressive and poorly differentiated tumors with higher Gleason scores. Our experiment demonstrated a strong visual PCa detection capability for C1, as well as high quantitative performance for F1 in distinguishing BPH from PCa and between intermediate and high-risk PCa. The diverse performance between C1 and F1 may be due to normalization process, which removes confounding factors but also reduces SNR and lesion contrast.

The **F2** outperformed other parameters in distinguishing between CS-PCa and CInS-PCa, as well as between low and intermediate-risk PCa, indicating that reducing in hindered diffusion or extracellular space may appear as low-risk PCa progresses.

It may be associated with the loss of organized glandular structures, increased stromal fibrosis, and reduced glandular lumen space, which are characteristic of higher-grade tumors where normal glandular architecture is largely replaced by disorganized cancerous growth and fibrotic tissue. F1F2 also exhibited similar comparable performance to F1 in distinguishing between intermediate and high-risk PCa. Other parameters such as C3, C4, F3, and F4, which represent free water and vessel flow components, may also serve as important biomarkers for tumor grading, although their utility is limited by low SNR, affecting their performance in differentiation.

The combination of two diffusion parameters from either a single model or two models significantly improved the AUC for differentiating between intermediate and high-risk PCa. Specifically, the combinations of MC C1 and F2, as well as FROC β and MC F1F2, outperformed other combinations or single parameters, suggesting that these models provide complementary information with low inter-parameter correlation. The MC model captures signal distribution from different tissue compartments, while the FROC model provides valuable insights into tissue heterogeneity [33].

In correlation analysis, parameters such as D, μ, C1, F1, and F1F2 were found to be highly correlated with ADC. This correlation may explain the high performance of these parameters in PCa grading, and the limited improvements in AUC observed in some comparisons. However, a few parameters, such as β and F2, showed low inter-parameter correlation and provided independent information to improve differentiation when combined.

In the visual assessment, ADC, D, and C1 exhibited good to excellent image quality, but only ADC and C1 demonstrated good to excellent prostate cancer detection capabilities. Although ADC and D offered higher overall image clarity than C1, the C1 parameter effectively suppressed benign tissue signals, enhancing tumor contrast for clearer lesion detection (Fig. 4), which aligns with previous studies [26, 27]. Other parameters exhibited sufficient to good image quality but lower PCa detection capabilities, which is differing from the statistical analysis. This may result from the limited sensitivity of the human visual system in detecting subtle quantitative differences. As shown in Fig. 2 and Table 1, the variations in F1 and F2 across different PCa levels were clear in the data but not easily seen by the human eye.

From the clinical practice perspective, the acquisition time for high and multiple b-values and the efficiency of algorithm implementations of the advanced models, could potentially limit their widespread usage. However, previous work demonstrated that the acquisition of MC model could be optimized within a 2-min scan on standard clinical scanners [27]. Moreover, the MC model’s algorithm is highly efficient, relying on linear fitting with only a few seconds processing. Thus, based on the acquisition and algorithm optimization, the advanced models show great potential in clinical application.

Our study has several limitations. Firstly, we did not include patient characteristics such as age, tumor location, and PSA levels due to the limited sample size, which constrained further stratification. Secondly, biopsy results were correlated with imaging findings based on the most probable location rather than using the gold standard of radical prostatectomy. Although biopsy-derived Gleason Scores are significant predictors of PCa mortality and are commonly used in risk stratification, this approach has its limitations. Thirdly, while our sample size is consistent with previous studies and reflects the actual distribution of the patient population [25, 34], increasing the sample size would enhance the robustness of our findings. Future research with a larger and more diverse cohort is needed to confirm these preliminary findings. Fourthly, the b-value range (0–2500 s/mm^2^) used in our study did not align with the typical FROC range, with maximum b-value up to 3000 s/mm^2^, commonly used in brain studies. However, as highlighted in review articles, a b-value of 2500 s/mm^2^ is generally considered high in prostate imaging. Lastly, other diffusion models such as diffusion kurtosis imaging (DKI) [17, 18] and intravoxel incoherent motion (IVIM) [19, 20] were not included, as they did not show added value compared to ADC in PCa evaluation according to previous studies [25, 35] and meta-analyses [36]. Emerging models like the diffusion-relaxation spectrum [37] and hybrid diffusion imaging [38] show great potential in correlating with prostate histology by separating tissue compartments, but were excluded due to the additional scan time required for various echo times.

In conclusion, this study systematically compared the performance of advanced diffusion models in differentiating prostate lesions. The multi-compartment model outperformed conventional ADC in all comparisons, particularly in distinguishing between low and intermediate, and between intermediate and high-risk PCa. Additionally, the combination of MC and FROC models further improved differentiation accuracy between intermediate and high-risk PCa. These advanced diffusion models hold significant potential for clinical use, enhancing diagnostic accuracy and the effectiveness of clinical decision-making.

## Data Availability

No datasets were generated or analysed during the current study.
